# Understanding the perceptions of risks versus the benefits of COVID-19 vaccinations in Manyara, Tanzania

**DOI:** 10.4102/jphia.v16i3.702

**Published:** 2025-04-18

**Authors:** Chima E. Onuekwe, Violet M. Mathenge, Alexander Makulilo, William Mwengee, Tumaini Haonga, Grace Saguti, Charles Sagoe-Moses

**Affiliations:** 1 Department of Immunizations, Emergency Preparedness and Response (EPR), World Health Organization, Dar es Salaam, United Republic of Tanzania; 2 Centre for Health and Allied Legal and Demographical Development, Research and Training (CHALADDRAT), Nnamdi Azikiwe University, Awka, Nigeria; 3 Department of Immunizations, Emergency Preparedness and Response (EPR), World Health Organization, Dodoma, United Republic of Tanzania; 4 Department of Political Science and Public Administration, Faculty of Social Sciences, University of Dar Es Salaam, Dar es Salaam, United Republic of Tanzania; 5 Arnold-Bergstraesser Institute, Freiburg, Germany; 6 Health Promotion Unit, Ministry of Health, Dodoma, United Republic of Tanzania; 7 Emergency Preparedness and Response (EPR), World Health Organization, Dar es Salaam, United Republic of Tanzania; 8 Department of Leadership and Management, World Health Organization, Dar es Salaam, United Republic of Tanzania

**Keywords:** COVID-19 vaccine, risk perception, vaccination, vaccine acceptance, hesitancy, Manyara region, Tanzania

## Abstract

**Background:**

Few would argue that vaccines have not saved more lives than any other medical invention. Eradicating smallpox, reducing wild poliovirus, saving the world from the Ebola virus, and reversing the trend of COVID-19 infections, vaccines are common references in revolutionising global health. These successes were not achieved without varying perceptions of the risks of diseases versus the benefits of vaccination.

**Aim:**

The study aimed to assess whether the perceived severity versus benefits of vaccination significantly influenced COVID-19 vaccination.

**Setting:**

This study was conducted in the Manyara region, Tanzania.

**Methods:**

A cross-sectional study were conducted among adults above 18 years across seven councils of the Manyara region, Tanzania. Forty focus group discussions (FGDs) and 32 key informant interviews (KIIs) generated qualitative data, in contrast to household surveys for quantitative data.

**Results:**

Over half, 54.6%, who perceived the COVID-19 vaccine as effective in preventing severe illness or death were vaccinated compared to those who disagreed (45.1%), indicating a strong association between vaccine effectiveness perception and vaccine acceptance or hesitancy (*p* = 0.003). Similarly, closeness or personal contact with an infected person was a major determinant for vaccination. Some 62.9% of respondents whose family members or friends were infected were vaccinated compared to 43.8% without any close experience of the infection (*p* = 0.032).

**Conclusion:**

This study found that perceptions of the severity of risks or benefits of vaccination significantly influenced the uptake of COVID-19 vaccination in Manyara.

**Contribution:**

This study underscores the importance of other factors that influence perceptions of risks and benefits of healthcare services even if they were offered at no cost to the beneficiary.

## Introduction

Since the first case of novel coronavirus later termed coronavirus disease 2019 (COVID-19) was confirmed in Wuhan, China in December 2019, the pandemic has caused major health, economic and social crises in most parts of the world. Forty-six out of 47 World Health Organization (WHO) member countries in the African region have reported outbreaks of varying proportions including Tanzania. Tanzania confirmed its first case on 16 March 2020. Since then, four waves of COVID-19 outbreaks have occurred with cases and deaths reported from all 31 regions.^1^ As of 14 February 2023, Tanzania confirmed 42 717 cases of COVID-19 virus including 846 deaths.^2,3^ Initial efforts to combat the pandemic primarily focused on non-pharmaceutical public health measures including regular handwashing, face-masking, and social distancing in all countries. Hence, Tanzania did not impose a complete lockdown of the country like other countries including East African neighbours. Rather, the national leadership of the country encouraged alternative traditional dietary remedies and steam inhalation [*kujifukiza*] against globally prescribed vaccines. This position was confirmed further on 02 February 2021, in Dodoma, Tanzania’s capital, when the health minister announced that Tanzania ‘has no plans in place to accept COVID-19 vaccines’. A few days later, President John Magufuli expressed doubt about COVID-19 vaccines sourced abroad insisting that Tanzania would only adopt vaccinations after they had been certified by Tanzania’s experts.^4^

With this position of the national leadership, the initial vaccine uptake was extremely low because of perceived low-risk and scepticism about the safety of the vaccine. Consequently, Konje, Basinda, Kapesa et al. published in September 2021 that two-thirds of Tanzanian healthcare professionals were hesitant about the COVID-19 vaccine and suggested it could be predictive of low uptake in the general population.^5^

Although before the outbreak of COVID-19, few would argue that vaccines have not saved more lives than any other medical invention. For example, eradicating smallpox, reducing wild poliovirus burden, saving the world from the Ebola virus, and reversing the trend of COVID-19 infections are common references.^6^ Continued success, however, depends on mass vaccination coverage, which in turn requires target beneficiaries to be committed to vaccination as an effective method of disease prevention. Such commitment is, however, affected by varying perceptions of the risks of diseases versus the benefits of vaccination. Understanding the factors influencing attitudes and perceptions towards the COVID-19 vaccine is critical, especially in the context of widespread rumours and conspiracy theories about the COVID-19 disease outbreak, vaccine development, deployment, and acceptance. People make decisions based not only on empirical data but various factors, including perceptions of severity, susceptibility, norms, personal experiences, socio-ecological effects, and more. Therefore, continuous understanding of the population’s perception of what is serious, what is risky, and what is best for someone’s health is imperative.^7^ Theoretical models of health beliefs, decision-making, and risk perceptions are critical for understanding the nuances behind acceptance or hesitancy to adopt health-related behaviours. Among well-documented theories and models of health behaviour, the health belief model (HBM) has remained the most popular and widely applied for examining the relationships between the perception of risk and output health behaviour.^10^ Importantly, HBM explains the complexity of intra-personal interactions before an individual adopts or adheres to a recommended health behaviour. The HBM principles include perceptions of the severity of the disease, perceptions of susceptibility to the disease weighed against perceptions of benefit to determine health behaviour, self-efficacy, perceived barriers, and cues to the recommended action. Several studies have shown that HBM is widely used to predict peoples’ vaccination behaviour.^8,9,10,11^ For example, Bish, Yardley, Nicoll, and Michie examined the psychological and demographic factors associated with the uptake of vaccination against Influenza.^12^ The study demonstrated the extensive use of the HBM to predict the perceived severity of Influenza and perceived susceptibility of getting inflected as predictors of the intention to get vaccinated.

Furthermore, previous studies by Shmueli and Yu, Lau, and She also explored perceptions of the risks or benefits of vaccination against COVID-19 to predict uptake and hesitancy. The studies showed a strong correlation between risk perception and vaccine acceptance.^13,14^ However, several studies have affirmed the willingness and/or intention of Tanzanians to be vaccinated against COVID-19. While Anjorin found that 71% of Tanzanians would accept COVID-19 vaccination, only a few visited any health facility for vaccination.^15^ The study showed that Tanzanians (91%) are knowledgeable about the dangers of COVID-19 and are willing to be vaccinated, yet few visited any health facility for COVID-19 vaccination. Vaccination was introduced lately compared to other neighbouring countries and public restrictions including lockdown were partially implemented. As a result, the perceived risk of COVID-19 infection was low. As of June 2022, the Manyara region in northern Tanzania had vaccination coverage of 3.7% and the lowest coverage among Tanzania’s 31 regions.^16^

World Health Organization’s recent review showed that Tanzania lacked data on COVID-19 vaccine acceptance rates, despite administering close to 36 million doses as of 20 February 2023. Other African countries with similar issues include Angola, Chad, and Mali.^17^ In addition, no studies have explored the effects of perceived risks of COVID-19 diseases and the benefits of vaccination in the Manyara region of Tanzania.

This study examined perceptions of the risks and benefits of vaccination, decision-making, and perceptions of severity and susceptibility to COVID-19 infection to predict the acceptance and hesitancy of COVID-19 vaccinations. The study aimed to use the findings to enable health authorities to predict vaccine acceptance and hesitancy in the future.

### Objectives

The objectives of the study are as follows:

- To examine whether the perceived severity of contracting COVID-19 could significantly influence vaccination uptake against the disease.
- To determine whether the perceived benefits of vaccination against COVID-19 to prevent illness or death from COVID-19 affect the uptake of vaccines among respondents who believe the vaccine is effective.
- To analyse whether personal experience or close contact with a COVID-19-infected person significantly influences uptake unlike among respondents who have not had a personal experience with COVID-19 infection or close contact with an infected person.

### Research questions

To achieve these objectives, we posed the following research questions (RQ):

**RQ1:** *Does the perceived severity of contracting COVID-19 significantly influence vaccination acceptance (risk perc eption)?*

**RQ2:** *Does the perceived benefit of vaccination against COVID-19 to prevent illness or death from COVID-19 affect the uptake of vaccines among respondents who believe that the vaccine is effective (perceived benefits of vaccination)?*

**RQ3:** *Doespe rsonal experience or close contact with a COVID-19-infected person significantly influence uptake unlike among respondents who have not had a personal experience with COVID-19 infection (subjective norm)?*

## Research methods and design

### Study site/population

The site for this study was Manyara, one of the 26 regions of Tanzania Mainland with an estimated population of 1.9 million across seven councils. Although Manyara had a fair share of 366 confirmed COVID-19 cases including 76 deaths as of December 2023, it was the lowest-performing region for vaccination against the disease as of July 2022. Hence, it became imperative to assess whether perceptions of the risks of COVID-19 or the benefits of immunisation impacted the uptake.

### Method of study

We conducted a cross-sectional study to generate qualitative and quantitative data among adults above 18 in the Manyara region. For qualitative data, 40 focus group discussions (FGDs) and 32 key informant interviews (KIIs) were conducted to collect relevant information from study participants on health beliefs, risk perceptions, and reasons for acceptance or hesitancy to COVID-19 vaccinations. The themes of the FGDs and KIIs include perceptions of the risks, and severity versus benefits of COVID-19 vaccination and reasons for acceptance or rejection of vaccination. The themes explored whether these reasons are connected to national leadership’s position on COVID-19 vaccines. The themes also explored whether participants believed in or practised traditional medicine as recommended by the health minister as a preventive measure against COVID-19. In contrast, quantitative data were generated through household surveys. Some 339 respondents were approached to participate in the survey. However, five respondents declined while 334 comprising 110 (33%) males and 224 (67%) females participated.

### Sample size and procedure

The sample size for this study was determined by an online Survey Monkey^18^ calculator. Manyara region has a target population of 105 570 above 18 years old. With a confidence level of 95% and a margin of error of 5%, an appropriate sample size was determined as 320 respondents. A multi-stage sampling procedure was adopted. The first stage was the selection of councils. Four councils (two rural and two urban) were selected. Manyara region has seven councils: two urban and five rural wards, namely Babati town council (TC), Babati district council (DC), Mbulu TC, Mbuliu DC, Hanang DC, Kiteto DC, and Simanjiro DC. Babati TC and Mbulu TC were sampled as urban councils while Mbulu DC and Kiteto were sampled as rural councils. In the second stage, two wards in each council (an urban and a rural one) were sampled while the third stage featured the sampling of two villages from each of the sampled wards. The ward centre was considered urban even in the rural ward while the second was considered as the rural village where the actual participants for the study were sampled. In each of the sampled wards, the Ward Executive Officer (WEO) provided the register of residents that formed the sampling frame for the study. The total registered residents determined the sampling interval of the participants until 20 participants were sampled per study unit. Every registered resident who met the criteria for participation was selected. If a sampled resident did not qualify, the next registered resident was considered until a qualified participant was selected. With the address on the register, the Village Executive Officer helped to reach out to the sampled respondents for participation in the FGD, KIIs, or household survey. If the sampled participant was unavailable or declined, the next sample was contacted until participation was confirmed.

### Data collection instrument

Forty FGDs across eight wards in four councils of the Manyara region generated qualitative data. For in-depth qualitative data, 32 KIIs were also conducted with selected community leaders, community health workers, religious leaders, leaders of community-based associations, and influential others. Using a set of questions as a guide, in-depth discussions were held on community perceptions of the risks and benefits of COVID-19 vaccinations.

A questionnaire was used to collect quantitative data from all participants. The questions were constructed in Swahili language to allow detailed understanding by the administrators and respondents. Socio-demographic as well as psycho-graphic variables were extracted from respondents. The first part of the questionnaire bordered on socio-demographic information that is, age, gender, education, income, dwelling, religion, and employment status. The second part was on COVID-19 vaccination status including reasons for acceptance or refusal of COVID-19 vaccination. The next part of the questionnaire assessed participants’ risk perception (severity, susceptibility, and barriers to COVID-19 vaccination). The questionnaire was pretested in Oldonyo and Osunyai wards of Arusha region between 17 September 2023 and 30 September 2023. The questionnaire was revised according to feedback from the pilot.

An Open Data Kit (ODK) was used to collect responses from the field. Data were uploaded immediately or as soon as the network was available. Three research assistants comprising national, regional, and council assistants trained on FGDs, KIIs, and household surveys facilitated the qualitative and quantitative data collection.

### Inclusion and exclusion criteria

For inclusion to participate in the study, eligibility criteria included all males and females above 18 years of age, who have resided in the council in the last 24 months, willing to participate in the study either as a key informant interviewee or FGD participant and must have the ability to speak and understand Swahili or English language. Samples who did not meet all the inclusion criteria were disqualified until a qualified participant was sampled.

### Ethical considerations

Ethical clearance to conduct this study was obtained from the University of Dodoma Research Review Ethics Committee (No. A.84/261/76/214). Before the commencement of the data collection, the purpose and significance of the study were explained to the Manyara Regional Health Management who granted the permission and communicated to the Council Health Management for permission to conduct the field study with the support of the Ward and Village Executive Officers. Informed consent was obtained from participants before joining the FGDs, KIIs, or household surveys.

## Results

### Socio-demographic characteristics of the study participants

A total of 339 respondents were approached to participate in the survey. However, five respondents declined while 334 comprising 110 (33%) males and 224 (67%) females participated. All participants were above 18 years old and had lived in the area for the last 24 months. The majority 287 (86%) have formal education while 47 (14%) have no formal education. Respondents were classified into three residential categories: urban, semi-urban, and rural dwellers. A total of 200 respondents, which is more than half of the respondents were self-employed, 94 (28%) had no employment, while 40 (12%) were employed by government, private or retired.

### Vaccination status across respondents’ socio-demographics

We assessed the vaccination status of respondents across socio-demographic variables (Table 1). Similarly, in previous studies by Neumann-Böhme, Varghese, Sabat et al. more men than women accepted vaccination.^19^ The majority of those who were vaccinated (53.5%), belong to the age group 60+. Supporting the finding, a key informant reaffirmed as follows:

‘When they said that old people are a priority for the vaccination against COVID-19, I knew that this is because our immunity is weak compared to young adults who feel strong and can withstand any infection.’ (Male, FGD participant, 65 years, Uhuru ward)

**TABLE 1:** Socio-demographic characteristics of the respondents across vaccination status.

Variable	Vaccination status of respondents according to socio-demographic variables						*p*
	Yes		No		Total		
	Frequency (*n*)	%	Frequency (*n*)	%	Frequency (*n*)	%	
**Age (years)**	-	-	-	-	-	-	0.060
18–29	20	32.7	41	67.2	61	-	-
30–39	55	51.4	52	48.6	107	-	-
40–49	33	47.1	37	52.9	70	-	-
50–59	21	39.6	32	60.4	53	-	-
60+	23	53.5	20	46.5	43	-	-
**Gender**	-	-	-	-	-	-	0.673
Male	65	47.8	70	52.2	136		-
Female	90	48.1	108	51.9	208		-
**Education**	-	-	-	-	-	-	0.218
No formal education	16	34.0	31	65.9	47	-	-
Primary education	100	48.8	105	51.2	205	-	-
Secondary education	28	43.1	37	56.9	65	-	-
University/tertiary	6	60.0	4	40.0	10	-	-
Vocational	5	71.4	2	28.6	7	-	-
**Employment status**	-	-	-	-	-	-	0.295
Government	11	57.9	8	42.1	19	-	-
Private	6	54.6	5	45.5	11	-	-
Retired	3	33.3	6	66.7	9	-	
Self-Employed	99	48.3	106	51.7	201	-	-
Unemployed	36	37.9	59	62.1	94	-	-
**Marital status**	-	-	-	-	-	-	0.414
Married	126	45.2	153	54.8	274	-	-
Single	13	39.4	20	60.6	33	-	-
Widowed	7	53.9	6	46.2	13	-	-
Divorced/separated	9	64.3	5	35.7	14	-	-
**Income level**	-	-	-	-	-	-	0.355
Above TZs 100k – TZs 249k	49	50.0	44	50.0	98	-	-
Above TZs 1m	4	66.7	2	33.3	6	-	-
Above TZs 250K – TZs 499k	13	33.3	26	66.7	39	-	-
Above TZs 500k – TZ 1m	6	40.0	9	60.0	15	-	-
Less than TZs 100k	83	47.1	93	52.8	176	-	-

**Total**	**155**	**45.7**	**179**	**54.3**	**334**	-	-

TZs, Tanzanian shilling; m, million; k, thousand.

Only 52.7% among participants aged 30–39 years, 47.1% for 40–49 years, and 21.3% for 18–29 years were vaccinated. More males, 47%, were vaccinated compared to 44.8% of the females. Understandably, women express more concerns about the possible side effects of vaccinations as it concerns fertility and breastfeeding. About 60% and 71% of respondents with university and vocational training were vaccinated, respectively, and only 34% of those with no formal education got vaccinated.

### Reasons for non-vaccination

Table 2 presents reasons for non-vaccination among survey respondents. Respondents who were not vaccinated were asked to state their reasons for not vaccinating. A majority (31.5%) said they had not been vaccinated because they doubted the safety of the COVID-19 vaccine.

**TABLE 2:** Reasons for not vaccinating. (*N* = 334)

Why were you not vaccinated?	Frequency (*n*)	%
COVID-19 is a scam	2	1.10
COVID-19 is not a problem	7	3.80
Doubt regarding the safety of COVID-19 vaccine	58	31.50
Friends/family members did not get the vaccine	1	0.50
No reason	43	20.00
Too busy	18	9.80
Other	50	27.20

**Total**	**179**	**100.00**

COVID-19, coronavirus disease 2019.

Fear of side effects after vaccination was expressed by the FGD participants and key informants as some of the reasons for non-compliance:

‘I cannot take the vaccine. After all, I am not well informed about its advantages and disadvantages because everything has advantages and disadvantages.’ (Male, key informant, 67 years, Palitinibo ward)

A female key informant also stated that safety concerns were some of the reasons she did not and could not convince anyone to accept vaccination:

‘Not only that I didn’t accept the COVID-19 vaccine, but I couldn’t convince anyone to accept it because I am not sure of the side effects now or later. There were a lot of rumours that the vaccinated would become infertile or die after a few years. Or do you want to talk about the short time when the vaccine was produced under emergency? No, no, no. I don’t trust the safety of the vaccine.’ (Female, key informant, 57 years, Dawari ward)

About 26% had no specific reasons for not vaccinating and the minority (0.5%) said they have not been vaccinated because their family members had not been vaccinated as well. However, 50% of respondents gave other reasons for not vaccinating including faith reasons (18%), being healthy (16%), and sickness or being pregnant at the time of vaccination.

Table 3 shows that respondents who perceived being at risk if not vaccinated had taken the vaccine (52.2%) while the majority (43.7%) who perceived no risk were not vaccinated.

**TABLE 3:** Perception of risk of contracting COVID-19 across vaccination status. (*N* = 334)

Do you feel at risk of contracting COVID-19?	Perception of risk of contracting COVID-19				Total	*p*
	Yes		No			
	Frequency (*n*)	%	Frequency (*n*)	%		
Yes	24	52.2	22	47.8	46	-
No	45	43.7	58	56.3	103	0.619
I don’t know	86	45.3	104	54.7	190	-

**Total**	**150**	**45.7**	**184**	**54.3**	**334**	-

COVID-19, coronavirus disease 2019.

### Perceptions of risk of contracting COVID-19

*The decision to vaccinate or not is complex, with perceptions of risks and effectiveness of vaccines, and the danger of diseases weighed against each other before a decision is made.* Understanding people’s perceptions of what can and cannot be controlled is important to understanding their behaviour. Table 5 shows the perceptions of the respondents on the effectiveness the COVID-19 vaccine.

### Perceived effectiveness of COVID-19 vaccine to prevent severe illness or death

Over half, 54.6% were vaccinated because they perceived the COVID-19 vaccine as effective in preventing severe illness compared to those who disagreed (45.1%) about the effectiveness of the vaccination. The results also indicated a strong association between the effectiveness perception and vaccination status (*p* = 0.003).

Corroborating the survey respondents, an FGD participant at Daudi ward also reaffirmed his perception of the effectiveness of COVID-19 vaccines in preventing severe illness:

‘I saw that those who took the vaccine were not getting sick. Even someone I knew who was always sick, got better after taking the vaccine, so I believed that it could prevent severe illness.’ (Male, 4-FGD participant, 39 years, Daudi ward)

### Personal experience with family members or close friends infected with COVID-19

Table 4 shows that 62.9% of respondents whose family members or friends were infected by COVID-19 were vaccinated as compared to those (43.8%) who did not have any close experience of the infection in their families or among friends.

**TABLE 4:** Personal experience of a family member or friend infected by COVID-19 across vaccination status. (*N* = 334)

Has any of your family members or friends been infected by COVID-19?	Yes		No		Total	*p*
	Frequency (*n*)	%	Frequency (*n*)	%		
Yes	22	62.9	13	37.1	35	-
No	133	43.8	171	56.3	304	0.032

**Total**	**150**	**45.7**	**184**	**54.3**	**334**	-

Participants at the FGDs and KIIs whose family members or close friends were infected by COVID-19 explained how they were influenced to accept vaccination by the experience:

‘When my husband came back from visiting a friend of his who got infected by COVID-19, he became sick too. So, I quickly took to the health facility for COVID-19 vaccination, though I did not plan to get vaccinated. Every other member of my family also got the jab as a result.’ (Female, FGD participant, 51 years, Babati ward)

## Discussion

This study examined risk perceptions, perceptions of the benefits of vaccination, and subjective norms as predictors of acceptance and hesitancy for COVID-19 vaccination in the Manyara region, Tanzania. We investigated socio-demographic characteristics including age, gender, education and income status, and health-related and behavioural variables. Vaccination uptake in older adults (those aged 70 years and over) was an initial focus of the global campaign. Hence, older adults were prioritised as a key group as increasing age is the leading risk factor for mortality and complications from COVID-19 infection. The main socio-demographic variable identified as a predictor of vaccination acceptance was age. The gender gap in COVID-19 vaccine acceptance results largely from women perceiving higher risks than benefits of the vaccines. Concerns about the safety of pregnancy, breastfeeding, and fertility among women may have skewed the gender difference in vaccination rate between men and women. As a group, older adults were most at risk of morbidity and mortality. So, it makes sense that more suffering will be relieved, and more lives will be saved if prioritised for vaccination. Our study found that most vaccinated (53.5%) were above 60 years. The finding is consistent with a similar study by Wang, Wong, Ho, and Cheung et al., which found a higher rate (93%) intention and 91.3% acceptance rate of vaccination among Chinese participants aged 65 years.^20^ Expectedly, older adults accepted vaccination more than other age groups. It is reasonable to find a higher acceptance of vaccination among respondents in this age group, as they are also included in the high-risk group for COVID-19. This was consistent with the initial campaign for prioritisation of older adults for vaccination, therefore, acceptance rate among older people was high. Aside from prioritising the older adults which created the perception of greater vulnerability, younger persons perceived COVID-19 vaccination differently. Firstly, the younger persons were more concerned about perceived side effects, especially on fertility than older adults who may not be productive anymore. Besides, they also believe that it was unnecessary for those at low-risk of harm from the virus to get vaccinated. Secondly, the younger participants were more exposed to social media content and as such influenced by negative social media narratives. Other socio-demographic variables were considered but no strong association was established between acceptance and refusal to vaccinate.

Earlier studies by Anjorin, Odetokun, Abioye et al. found 71% acceptability and 63% willingness to receive COVID-19 vaccination among Tanzanians, consistent with studies elsewhere in British Columbia, Canada, and Israel (Ogilvie, Gordon, Smith).^21,22^

Before the acceptance of the COVID-19 vaccination in Tanzania, various political and religious leaders had made critical public statements on the safety and efficacy of COVID-19 vaccine to prevent illness associated with the disease. Hence, the population was thrown into a crisis of perception. Safety concerns contributed 31.1% and were the major reason for hesitancy. In earlier studies, concerns about the safety of the vaccines were a major reason for hesitancy to vaccinate. Concerns about the safety of COVID-19 vaccine may be because of several reasons although not limited to the speed at which the vaccines were developed, the controversies that heralded the pandemic, and reported side effects, such as headaches, fatigue, and muscle pain associated with COVID-19 vaccines. Hence, it is imperative to strengthen public health education campaigns on the safety of vaccines to convince people to accept the vaccination.

### RQ1: Does the perceived severity of contracting COVID-19 significantly influence vaccination acceptance (risk perception)?

The study questioned whether the perceived severity of contracting COVID-19 could significantly influence vaccination acceptance. Confirmatively, we found that more than half of the respondents (56.3%) who did not perceive themselves as being at risk were not vaccinated. This finding affirms our first research question that the perceived severity of contracting COVID-19 disease significantly influences vaccination acceptance. Other studies (Nusair, Arabyat Khasawneh et al. and Robertson, Mohr, Barjaková et al.) also indicated that individuals who perceive that they have a higher likelihood of experiencing complications from COVID-19 have a higher acceptance of vaccination than others.^23,24^ Impliedly, when a person has a low-risk perception about the severity of a disease, he may not accept vaccination against the disease.

### RQ2: Does the perceived benefit of vaccination against COVID-19 to prevent illness or death from COVID-19 affect the uptake of vaccines among respondents who believe that the vaccine is effective (perceived benefits of vaccination)?

Furthermore, this study found a strong correlation between the perceived benefit of vaccination against COVID-19 and acceptance of the vaccination. This finding answered our second question in the affirmative. The perception that vaccination is effective in preventing illness and death related to COVID-19 infection has influenced acceptance (see Table 5). Also, our finding is consistent with other studies (Benham, Atabati, Oxoby et al. and Wang, Chukwu, Mwanyika-Sando et al.) in which most survey respondents and interview participants who intended to receive a COVID-19 agreed that COVID-19 disease was severe and vaccination was necessary.^25,26^ Furthermore, the study by Wang et al. on the acceptability of other vaccines linked acceptability to the perceived effectiveness and importance of vaccination.^27^

**TABLE 5:** Perceived effectiveness of COVID-19 vaccine across vaccination status. (*N* = 334)

Do you believe that COVID-19 vaccines are effective in preventing severe illness?	Yes		No		Total		*p*
	Frequency (*n*)	%	Frequency (*n*)	%	Frequency (*n*)	%	
Agree	90	54.6	75	45.5	165	-	-
Disagree	23	45.1	28	54.9	51	-	0.003
Neutral	42	34.2	81	65.9	123	-	-

**Total**	**150**	**45.7**	**184**	**54.3**	**334**	-	-

### RQ3: Does personal experience or close contact with a COVID-19-infected person significantly influence uptake unlike among respondents who have not had a personal experience with COVID-19 infection (subjective norm)?

We also asked whether personal experience or close contact with a COVID-19-infected person could significantly influence vaccination. Our finding answered the question affirmatively as 62.9% of the vaccinated persons have had a personal or close experience with an infected family member or friend. Consistent with other studies elsewhere, Akel found similar results in a study on the impact of personal contact or close experience vaccination intention across six countries including the United States (US), India, Indonesia, and China.^27^ Overall, the study reported that COVID-19 vaccine acceptance was higher among those with serious and personal experiences with COVID-19 in most locations. The study found that in the US, participants with serious personal experiences were nearly 40% more likely to accept a COVID-19 vaccine than those with no experience. On the contrary, exposure to serious cases in the media in Indonesia had a higher impact on vaccine acceptance.

## Conclusion

The study examined perceptions of the risks of diseases to enable prediction of the acceptance or hesitancy of COVID-19 vaccinations. Decision-making is influenced not only by empirical data but also by individuals’ health beliefs, norms, perception of self-efficacy, personal experiences, and social pressure for approval or belongingness. Our findings are in line with studies from other countries, which have shown that, among others, perception of risks and benefits of vaccination, concerns about vaccine side effects and safety, and the hitherto unfavourable position of the national government of Tanzania about COVID-19 contributed to the low uptake. The disparity in vaccination rates based on socio-demographic variables, especially age and gender, confirms the importance of considering those factors while planning public health interventions. The prioritisation of older adults above 60 years as a vulnerable population undoubtedly influenced the acceptance of vaccination by the subjects more than any other group. On the other hand, women’s concerns about the safety of pregnancy and fertility-related matters while considering acceptance of vaccinations also affected uptake by the female population. Therefore, there is a need for continuous understanding and interpretation of the population’s perception of what is serious, what is risky, and what is best for their health.

By applying qualitative and quantitative methods, this article argued that understanding the perceptions of risks of diseases through the synthesis of health belief models, decision-making, and subjective risk perception could predict vaccine behaviours. The findings of this study are important for public health policymakers and healthcare providers to predict vaccination behaviour. Efforts should concentrate on raising risk perceptions of the severity of diseases and the benefits of vaccination. It is equally imperative to encourage individuals to share their experiences regarding COVID-19 vaccination on public media.

An effective behaviour change strategy for COVID-19 vaccinations is needed to address perceptions of the risks of COVID-19 disease and the benefits of vaccinations and leverage enablers identified in this study to increase acceptance. Further research should expand the scope of similar studies to other regions to extrapolate the results conveniently beyond one region.
